# Diabetes—the Effect of Climate in Its Treatment

**Published:** 1845-11

**Authors:** K. Imray


*Diabetes—the Effect of Climate in its Treatment.* By K. Imray, M.D.—Perhaps the treatment of diabetes by an animal diet has met with more success, at least as a palliative, than any other—and it would probably be more successful if it could be rigidly followed up, which is next to an impossibility. The use of opium is another useful palliative. Diaphoretics are especially indicated by the dry, parched skin, which so generally exists, and which is often one of the earliest symptoms. A return of the natural functions of the skin is also one of the earliest symptoms of amendment. This has been remarked, many years ago, by Dr. Christie, who found the disease much more manageable in Ceylon than in this country. It is, moreover, seldom seen in very warm climates. Dr. William Hunter, during a long residence in Bengal, never saw a case; and Dr. Imray, during a residence of seven years in the West Indies, never saw or heard of a case commencing there. From several cases which this gentleman has published, we should say that a residence in a very warm climate is a most powerful means of restoring the healthy function of the skin, and curing the disease.—*Edinburgh Med. and Surg. Jour.—Ibid.*

---

*Efficacy of Liquor Ammonia in Pertussis.*—Dr. Peyroton recommends the following prescription in whooping cough—

℞ Aq. distil. lactucæ vir. ℥iv.

——— flor. aurant. ʒij.

Syrup. pæoniæ officin. ʒj.

——— belladonnæ ʒij.

Ammon. liquor. qts- vj.

Misce, ft. Mist. coch. magn. unum 4tis horis sumendum.

Four cases are quoted as proofs of its efficacy; the cure in all was rapid and complete.—*Revue Médicale.—Medical Times.—Ranking's Abstract.*

